# Kinetic Control of Parallel versus Antiparallel Amyloid Aggregation via Shape of the Growing Aggregate

**DOI:** 10.1038/s41598-019-52238-x

**Published:** 2019-11-05

**Authors:** Ali Asghar Hakami Zanjani, Nicholas P. Reynolds, Afang Zhang, Tanja Schilling, Raffaele Mezzenga, Joshua T. Berryman

**Affiliations:** 1 University of Luxembourg, Department of Physics and Materials Science, 162a Avenue de la Fäıencerie, Luxembourg City, L-1511 Luxembourg; 2 Swinburne University of Technology, ARC Training Centre for Biodevices, John Street, Melbourne, VIC, 3122 Australia; 3 Shanghai University Department of Polymer Materials, Nanchen Street 333, Shanghai, 200444 China; 4 Institute of Physics, University of Freiburg, Hermann-Herder-Str. 3, 79104 Freiburg im Breisgau, Germany; 5 Department of Health Sciences and Technology, ETH Zurich, CH-8092 Zurich, Switzerland; 6 Department of Materials, ETH Zurich, Wolfgang-Pauli-Strasse 10, 8093 Zurich, Switzerland

**Keywords:** Prions, Chemical physics, Biochemical reaction networks

## Abstract

By combining atomistic and higher-level modelling with solution X-ray diffraction we analyse self-assembly pathways for the IFQINS hexapeptide, a bio-relevant amyloid former derived from human lysozyme. We verify that (at least) two metastable polymorphic structures exist for this system which are substantially different at the atomistic scale, and compare the conditions under which they are kinetically accessible. We further examine the higher-level polymorphism for these systems at the nanometre to micrometre scales, which is manifested in kinetic differences and in shape differences between structures instead of or as well as differences in the small-scale contact topology. Any future design of structure based inhibitors of the IFQINS steric zipper, or of close homologues such as TFQINS which are likely to have similar structures, should take account of this polymorphic assembly.

## Introduction

The hydrogen-bonding, hydrophobic and electrostatic interactions which stabilise globular proteins can also drive the formation of tough multi-chain ‘amyloid’ aggregates which are often associated in biology with disease^1,2^. Amyloid formation is implicated in various pathologies, particularly fatal neurodegenerative diseases such as Alzheimer’s, Parkinson’s and Huntington’s^3–6^. Beyond the neurodegenerative diseases, certain inherited amyloidoses may be systemic or else localised in non-brain tissues: lysozyme amyloidosis is an example of this class, in which a mutation in the IFQINS subsequence (to TFQINS) leads to accumulation of amyloid and eventual multiple organ failure^7,8^. A further relevant sequence variation is I**L**QINS, the wild type subsequence in *Gallus gallus*, which demonstrates reduced *in vitro* amyloid formation relative to the human wild type IFQINS^9^.

In the study of amyloid aggregation it is common to use truncations or subsequences of longer bio-relevant proteins, in some cases because the protein is indeed truncated in the biological context but often also for simple convenience. The (I/T)(L/F)QINS peptide system has elements of both of these motivations: it was shown using mass spectrometry that in conditions of warm acid similar to the stomach, full-length lysozyme is hydrolysed into fragments, and that fragments containing (I/T)(L/F)QINS such as Y_54_G**ILQINS**RWWCND_67_ dominate the aggregation process^9^. As well as I56T, we should note that W64R and D67H are disease-associated mutations^10^, nevertheless in order to develop full understanding and control of the aggregation process we focus currently on the 6-residue fragment only. It has been shown that aggregation propensity increases following the sequence *ILQINS* → *IFQINS* → *TFQINS*, and *pH*7 → *pH*2^11^. In the same paper, a computational prediction that amyloid formation should in some cases *decrease* with increasing concentration was made, and validated experimentally. The decrease of total precipitate mass in this case was associated to a greater proportion of helical-ribbon fibrils, and a lesser proportion of rectangular microcrystal or rod-like fibrils. We should remark that this previous paper used different candidate atomistic structures for the hexapeptide systems but that, as the mutation series only alters sidechains at the unit-cell surface, conclusions for self-assembly from that study remain qualitatively unchanged when repeated using the newer candidate atomistic structures.

Analysis of short peptide steric zippers has in the past led to successful design of inhibitors for aggregation of the full-length chain, including aggregation of the A*β*^12^ and tau peptides^13^. Tau includes the VQIVYK and VQIINK homologue hexapeptides of IFQINS, and effectiveness of inhibitor design was improved by targeting the polymorphic steric zippers for VQIVYK and VQIINK^14^, including structural information from soluble nanocrystal or fibril structures as well as from microcrysytals amenable to solid-phase crystallography. Effective design of inhibitors for human lysozyme aggregation should therefore also benefit from understanding of IFQINS polymorphic steric zippers. The kinetic process by which polymorphs compete or cross-seed can potentially be complex. If amyloid aggregates propagating as prions are a form of highly simplified quasi-lifeform, then this network of polymorph interactions is the quasi-ecology which determines dominance or extinction of a given fold.

Research into amyloid is not only driven by medical goals, but also seeks to develop peptide biomaterials^15–18^. One of the motivations to consider amyloid as a biomaterial is the potential for versatility in material properties driven by polymorphism at the atomistic or mesoscopic levels: it is common that a given sequence can stably take on a variety of morphologies^19^ including filaments^20^, nanotubes^21^, helical ribbons^9,11,22^, twisted ribbons^11,22^ and crystals^11,23^ depending on the growth conditions.

Here, we examine solution scattering taken during the aggregation process at high peptide concentration in water which shows an aggregated structure for IFQINS that is consistent with a solid-phase crystal structure previously published (by Sievers *et al*.^24^, pdb code **4R0P**^25^), but which is different to the solution scattering previously observed. We show that the medium-concentration structures previously studied are composed of antiparallel (AP) *β* sheet, while the crystal and higher-concentration solution are composed of parallel (P) *β* sheet. The experimental data contrasting these two polymorphic structures which differ in the symmetry of assembly allows us to extend our modelling of the hexapeptide aggregation process and examine the physics of selection between polymorphs formed from P and AP *β* sheet.

## Results

### Atomistic simulations compared to WAXS

**Figure 1:**
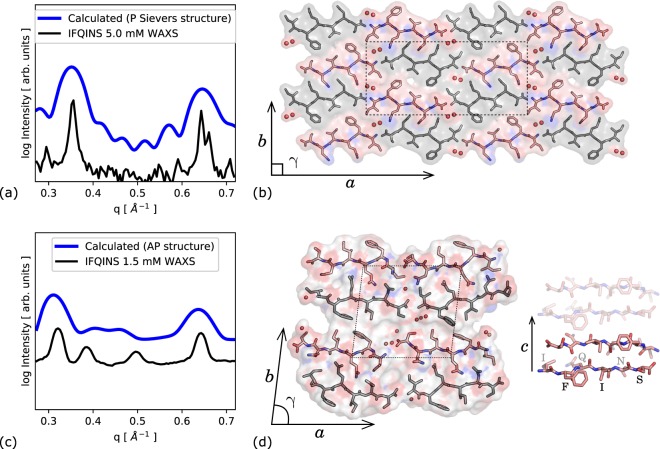
(**a**) Calculated scattering based on solid-phase crystal structure after evolution in aqueous environment for 15 ns. All curves are scaled such that the single highest peak is 1 (experiment) or 5 (simulation); scaling is consistent between both calculated curves. (**b**) Solid-phase P crystal structure **4R0P** of IFQINS (four unit cells) reported by Sievers; lattice parameters are *a* = 43.2 Å, *b* = 19.6 Å and *γ* = 90°. A full translational unit cell is shown as a rectangle in the center. Crystalline waters are indicated as red spheres. (**c**) Calculated scattering curve based on computationally derived AP structure, compared to solution scattering; unit cell is *a* = 20.0 Å, *b* = 19.1 Å and *γ* = 83°. (**d**) Class 5 AP structure, with semi-ordered dynamic water molecules shown as spheres. The *c*-axis is the inter-peptide hydrogen bonding direction, or ‘fibril’ axis. Sticks show the AP stacking geometry along *c*, fading indicates translation to an equivalent peptide pair on the opposite side of the steric zipper. [Graphics produced with pymol 1.8 http://pymol.org and inkscape 0.91 http://inkscape.org].

Atomistic models of the **4R0P** parallel-*β* crystal structure and a designed AP-*β* structure were placed in a virtual aqueous environment and allowed to relax for 15 ns (see methods), and calculated scattering was compared to WAXS spectra collected from real solutions with high (5 mM) and low (1.5 mM) concentrations of peptide. The lower-concentration experimental scattering agrees quite well with calculations based on the designed AP structure (Fig. 1(c,d)), while the X-ray data for a high-concentration (5 mM) solution of IFQINS after 24 h agrees with scattering calculated based on the **4R0P** deposited crystal structure (Fig. 1(a,b)). Despite the differing symmetry of **4R0P** to the AP structure, the overall scattering is not completely dissimilar, however the 180° rotation which accompanies translation along the *a*-axis in **4R0P** leads to fewer (but not much shifted) peaks in the angular window considered than were observed from IFQINS aggregated at lower concentrations. Peaks from the **4R0P** structure are much sharper than from the AP structure, both in experiment and simulation, indicating stronger ordering.

While the calculated solution-phase scattering for the P structure is based on solid-phase crystallography carried out to a high resolution, the AP structure was developed for comparison to the solution scattering rather than being directly fitted to it as the solution scattering curve contains insufficient information to usefully guide a fit. Additionally, the forward calculation of scattering from structure is quite cumbersome in the solution case as the model aggregate is not constrained to be space-filling, therefore a large supercell-aggregate with whatever twist or other deformation naturally emerges from the modelling must be used, together with a quantity of explicit water to capture solvation structure and solvent interpenetration. Only one test simulation and forward calculation of solution scattering (see methods) was therefore made. The two peaks which do most to distinguish the two sets of experimental scattering (between 0.35 and 0.55 Å^−1^, Fig. 1(a,c)) are respectively absent and present in the P and AP calculated scattering. These two peaks were previously suggested to be related to combinations of the *a* and *b* lattice vectors, i.e. to have the first two Miller indices as both non-zero^9^, and this is consistent with being absent in the **4R0P** structure given that (for instance) no vector comparable to the 110 vector of the AP structure is a translational symmetry in the **4R0P** structure. If the two imperfectly-fitted peaks indeed correspond to the 110 and related translations in the AP structure then they are dependent on the angle *γ* (unlike the two larger peaks, assigned to unmixed multiples of *a*, *b* and *c* lattice vectors). This *γ* was previously shown to be a quite soft degree of freedom for model fibrils like the designed AP structure, however the extra rotation accompanying translation about *a* in the **4R0P** constrains *γ* to be 90° for this system.

### Peptide-level assembly thermodynamics

Having arrived at two atomistic models for solution nanocrystals, we compare them by evaluating standard free energies to join together the different lattice planes of the nanocrystals under a linear approximation such that the total energy scales proportionally to the number of peptides buried by the interface. We find the free energy per peptide for interfaces perpendicular to the *a* (terminus-terminus axis), *b* (sidechain interaction axis) and *c* (hydrogen bonding axis) by calculating the difference between free energies of joined blocks of peptides and separated blocks, for example:

$$\Delta {G}_{a}^{\circ }=({G}_{243}-2{G}_{143})/(4\times 3),$$

where integer subscript triplets *ijk* are the number of peptides in each dimension of a rectangular peptide block or sub-block, and the denominator term is the number of peptides buried in the reference interface. The free energy to form a steric zipper, creating an interface which cuts through a unit cell of the crystal lattice, was also calculated. This interface, Δ*G*_*zip*_, is parallel to the *b* lattice plane, but is stronger than Δ*G*_*b*_. Where a splitting event changes between a single block with even *j* and two blocks with odd *j*, Δ*G*_*zip*_ is relevant rather than Δ*G*_*b*_. Conversely when a splitting event changes moves from an even-numbered *j* (a single aggregate with an even numbered count of peptides along the sidechain axis *b*) to two smaller even *j*, the fracture is treated as having occured on the *b* plane rather than the intra-lattice *zip* plane. This removes from consideration a set of somewhat-pathological ‘inside-out’ species having a broken steric zipper on the outside but a weaker *b* interface buried.

Reference block free energies *G*_*ijk*_ are calculated as averages over 100 blocks sampled from the converged part of the MD simulation. After a block is ‘cut’ from the simulation system, its energy is minimised in a continuum solvent^26^, so that the final free energy accounts for the electrostatics of solvent exposure, and also contains part of the appropriate physical entropy change from creating an interface, particularly that related to ordering of the solvent.

**Figure 2:**
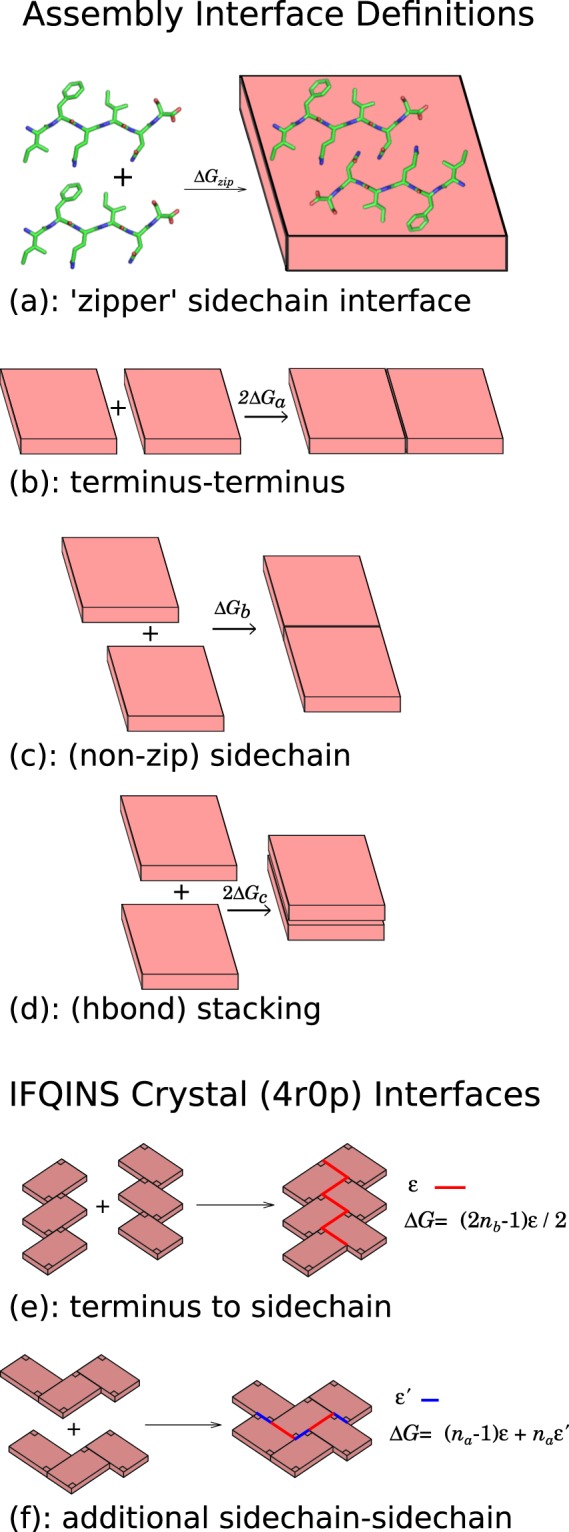
Cartoon showing binding free energies for interfaces in the *a* (backbone axis), *b* (sidechain axis) and *c* (hydrogen bond axis). **(a**–**d)** AP structure has generic in-register steric zipper 3D assembly. **(e**–**f)** Sievers’ structure has herringbone assembly characterised by two energy terms *ε* and *ε*′. In Sievers’ structure the binding free energies in *a* and *b* directions are **(e)** Δ*G* = (2*n*_*b*_−1)*ε*/2 and **(f)** Δ*G* = (*n*_*a*_−1)*ε* + *n*_*a*_*ε*′, where *n*_*a*_ and *n*_*b*_ are the number of peptides in *a* and *b* directions, respectively. [Graphics produced with pymol 1.8 http://pymol.org and inkscape 0.91 http://inkscape.org].

Because the **4R0P** structure has a herringbone symmetry (group *p*2 in the *ab* plane) rather than pure translational (group *p*1), the edges of an assembly are jagged with substantial overhang, and writing the free energy to join two blocks as a straightforward linear sum is less appropriate than for the AP structure (Fig. 2). As well as these edge irregularities visible in projections onto the *ab* plane, adjacent sheets are also stepped by ±0.5*c* in the vertical *c* axis, so in general the calculated interface energy based on the *ab* plane should be multiplied by *n*_*c*_ − 1/2 rather than by *n*_*c*_ as is the case for strictly rectangular blocks. Figure 2e,f gives definitions for two components of the interface energy, which we call *ε* and *ε*′, that can be used to compose the binding free energies in the *a* direction as:

1 $$\Delta G/({n}_{c}-1/2)=(2{n}_{b}-1)\varepsilon /2$$

and in the *b* direction as:

2 $$\Delta G/({n}_{c}-1/2)=({n}_{a}-1)\varepsilon +{n}_{a}\varepsilon ^{\prime} $$

where *n*_*a*_, *n*_*b*_, *n*_*c*_ are the numbers of peptides in the *a*, *b*, *c* directions respectively. The expression (2*n*_*b*_ − 1)*ε*/2 for the energy per *a* interface was chosen to be reasonable in the limit of single-sheet association (*n*_*b*_ = 1 implies half a steric zipper, and gives *ε*/2 desolvation energy) and also for the subsequent addition of whole steric zippers, such that adding 2*n*_*b*_ adds 2*ε*.

Given the stronger surface penalty for the P lattice relative to the AP lattice, there seems to be an immediate qualitative explanation for the **4R0P** structure to have a higher nucleation barrier than the AP structure, and therefore for it to form later (or never) under conditions of lower concentration where nucleation or meta-nucleation is a more significant limit to the aggregation process, however we will also discuss other differences between the two systems.

The standard binding free energy gains to construct a buried interface associated with each cleavage plane Δ*G*_*a*_°, Δ*G*_*b*_°, Δ*G*_*zip*_° and Δ*G*_*c*_° are written in Table 1 in kcal/mol/peptide. The parameters *ε* = −18.84 kcal mol^−1^, and *ε*′ = −5.99 kcal mol^−1^ were found in the same way as the others, by comparing blocks of peptides (see Methods). Although the lattice of **4R0P** is not directly comparable to that of the AP structure, in the limit of large microcrystals addition of a new interface in *a* will add an energy close to 2*n*_*b*_*ε*/2 (compared to 2*n*_*b*_Δ*G*_*a*_° for the AP structure), and a similar limit exists for growth in *b* (Fig. 2). These large-aggregate limits are shown in the table in order to highlight that lateral growth is both stronger and more isotropic for the **4R0P** structure once an initial nucleation barrier (or a complex nucleation-like kinetic bottleneck) has been passed. At an atomistic level in the specific case documented here, this isotropic quality arises partly from the hydrogen bonds between termini and side-chains (Fig. 1(b)), ‘mixing’ sidechain and terminus-driven assembly. In general P-*β* sheet formed of identical peptides allows a smoother sheet surface than AP (through stacking of like sidechains)^27^, this should lead to the phenomenon of more isotropic assembly for P rather than AP amyloid being widespread.

**Table 1:** Standard binding free energy gain to construct a buried interface in the direction of backbone axis (Δ*G*_*a*_°), sidechain axis (Δ*G*_*b*_°), intra-cell zipper (Δ*G*_*zip*_°, in the plane perpendicular to the *b* axis), and hydrogen bond axis (Δ*G*_*c*_°).

IFQINS Structure	Δ*G*_*a*_°	Δ*G*_*b*_°	Δ*G*_*zip*_°	Δ*G*_*c*_°
designed AP class 5	−9.2 (1)	−6.2 (1)	−16.4 (3)	−29.3 (2)
4R0P crystal class 1	*ε*/2 = −9.4 (1)	*ε* + *ε*′ = −24.8 (2)	−25.3 (2)	−25.3 (2)

For the crystal structure, energies are initially not linear with *n*_*a*_, *n*_*b*_, *n*_*c*_ however the linear change per increase in dimension at the limit of large aggregates is shown. Units are kcal/mol/peptide buried by the interface. Parentheses indicate the statistical uncertainty (ESE) in the final digit.

### Complex kinetic competition

**Figure 3:**
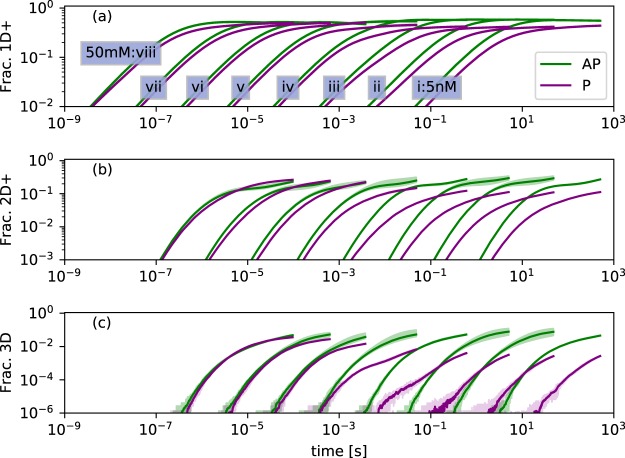
Evolution of microcrystal formation colored by parallel or antiparallel. Curves i–viii show concentrations increasing from 5 nM in multiples of 10. Spreads show minimum and maximum values reached in 10 replicates. Traces are averages over the replicates. ‘Frac. 1D+’ (**a**) shows peptides incorporated into any aggregate which is identifiably P or AP, therefore initially describes mostly single-sheet aggregates, for which AP is the most stable geometry. (**b**) shows formation of 2D aggregates, where complex kinetics driven by the availability of 1D aggregates and the stability of 2D+ aggregates begin to take effect. (**c**) shows the quite sharp kinetically-driven phase transition between AP dominance and AP-P coexistence in the final sample.

Having identified selection *in vitro* between two dissimilar structures, the event-driven Gillespie algorithm was used to make a kinetic simulation investigating the competition between the AP and P *β*-sheet structures over a range of concentrations. Figure 3 shows the evolving mass of aggregated peptides, broken down by elongation (Fig. 3(a)), then formation of 2D and 3D aggregates (Fig. 3(b,c)). A complex kinetic with two regimes is evident, at low and high concentrations.

At low concentrations (nM-mM) the AP structure elongates noticably faster, as the weaker Δ*G*_*c*_° for P *β*-sheet makes the formation of single-sheet aggregates reversible for these structures on timescales approximating that of collisions. The lead of AP in forming 1D aggregates translates into formation of 2D and 3D aggregates by hierarchical self-assembly and the AP system dominates assembly at lower concentrations. The small amount of P assembly which does take place in this regime shows a stochastic distribution of wait times, indicating that rare nucleation events are needed for highly stable 3D P aggregates to form. At high concentrations (mM and up), the gain of the AP system in forming 1D aggregates is overtaken by the P system in forming 2D aggregates: the stronger steric zipper and lateral assembly in the P geometry allows it to form 2D structures with long-term stability while the pool of free monomers is still not fully depleted. At these higher concentrations the proportion of 1D or higher AP aggregated peptide even takes a gradual downward trend, as peptides leave the 1D+ AP aggregates and are recruited into 2D and higher P aggregates. Within the simulation timescale, dominance of P over AP is never dramatic. The turnover at which the two are roughly equal is located at around 5 mM, the concentration at which a mixed population of fibrils was observed experimentally.

The mesoscopic shape of the growing aggregates (twist, bend and aspect ratio) has an important connection to the kinetics, in that bent and twisted aggregates have reduced possibilities for hierarchical self assembly without paying an energetic penalty to un-twist or un-bend. In the physical system twist and bend are coupled to cross-section area and aspect ratio, with smaller area implying less cost to twist, and an aspect ratio further from one (large *N*_*a*_/*N*_*b*_ or large *N*_*b*_/*N*_*a*_) implying less cost to bend^28^, although the relationship may be complex. Elastic deformation was not treated directly in the models presented here, however we find that the turnover in empirically observed aggregation at the 1.5 mM to 5 mM range of concentrations corresponds to an inflection in the aspect ratio behaviour for AP aggregates (Fig. 5).

**Figure 4:**
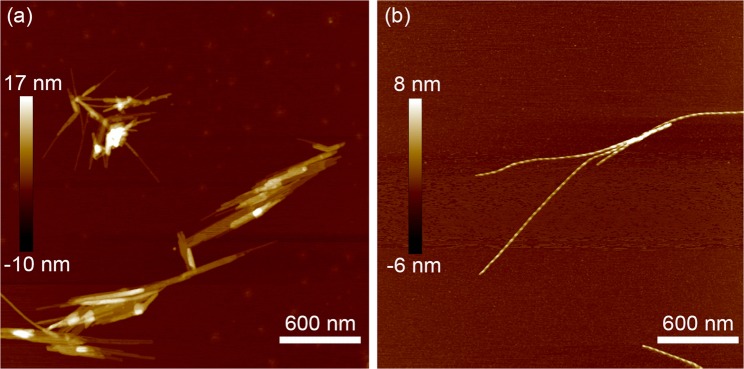
Height-mode AFM images for IFQINS samples after 24 h assembly in water at **(a)** 1.5 mM and **(b)** 5.0 mM. The higher-concentration samples produced more helical ribbons, and less total mass of deposited aggregate.

Previous experiment and modelling observed an increase in aspect ratio moving from 1.5 mM to 5 mM concentration (in multiple sequences, at multiple pH values), and explained a reduction of large aggregates, particularly of large rectangular aggregates, as resulting from the increased tendency to twist and to curl into helices of large aspect-ratio aggregates^11^. This effect is illustrated by AFM imaging in Fig. 4. The kinetic arrest on the pathway to the global free energy minimum (of large amyloid-like crystals) remains a feature in the new modelling, based on an improved AP computational structure and also including the crystallographic P structure, however it is not at this stage obvious how much of the kinetic is determined by this meso-polymorphism and how much by the P versus AP competition which is the main novelty of the present work. That the effect (on the computational structure) has the same sign in either version of the modelling, with or without P/AP polymorph competition, is evidence that the aspect-ratio-driven kinetic competition goes on independently of the P/AP competition. The crystallographic P structure shows very little deviation from 1 in its aspect ratio: probably a major reason that it was possible to grow micron-scale crystals in this conformation.

**Figure 5:**
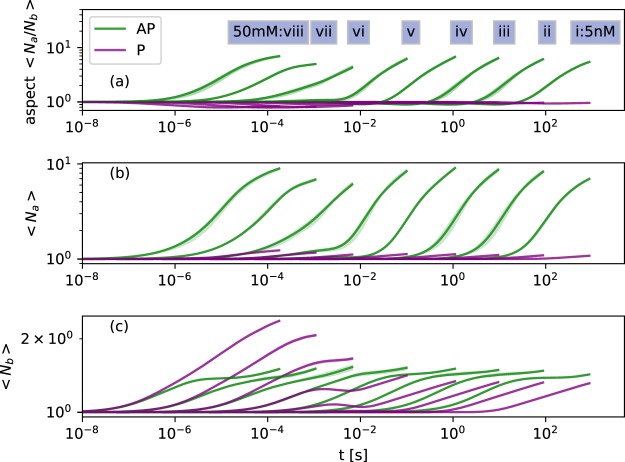
Evolution of cross-sectional aspect ratio colored by parallel or antiparallel. Monomers are counted as both P and AP for purposes of averaging. Spreads show minima and maxima reached in 10 replicates. Traces are averages over the replicates. Both P and AP show complex behaviour crossing from *μ*M to mM concentration. The formation of the first steric zipper occurs at *N*_*b*_ = 2, stabilisation or slowed growth of <*N*_*b*_> between 1 and 2 indicates that many 1D or 2D aggregates remain in solution.

At *μ*M concentrations and below, the AP aspect ratio *N*_*a*_/*N*_*b*_ initially drops very slightly, driven by steric zipper formation parallel to the *b* lattice plane (*N*_*b*_ = 2 implies a steric zipper has been formed). The non-zipper hydrophobic *b* interface is however less stable than the terminus-terminus *a* interface (Table 1), so the more stable *a* interface then takes over and leads growth, driving formation of large quasi-2D sheet pairs which are likely to become helical ribbons or twisted fibrils in the physical system. Above mM concentrations, *a* and *b* interfaces are both stable on the timescales of oligomer collision, and the aspect ratios do not run away to the same extent. Based on aspect ratios, either very low or very high concentrations emerge as optimal for formation of more rectangular, less twisted nanocrystals or thick fibrils in the AP geometry.

The P aspect ratio trace shows that at low concentrations the P system is reliant on formation of a 3D aggregate for stability, so has slow but roughly isotropic growth following the initial formation of the steric zipper such that *N*_*b*_ = 2. At high concentrations (where P aggregates are observed empirically), growth in *b* is enough to stabilise the P system without much growth in *a*, so this axis leads the lateral growth but not so much as to cause dramatic anisotropy.

The crystallisation experiment leading to P-*β* structures used a buffer solution not modelled in simulation, however we note that it took place *via* hanging drop method from an initial concentration of 5 mM^24^, the region where P formation is also strong in the simulation. The WAXS experiments took place in pure water, and produced either pure AP fibrils or a mixture of P and AP.

## Discussion

Here we examine a system with kinetic competition between parallel and antiparallel aggregation (P/AP), and show a somewhat counterintuitive pattern whereby the structure with a smaller free energy of formation per unit volume (AP) is nonetheless favoured, particularly at low concentrations, due to having no single high barrier in its metanucleation pathway.

In general, P and AP sheets contrast in that AP systems have stronger axial interactions in the direction of the *β*-sheet, while P systems (especially those which are antiparallel across the steric zipper interface) can compensate for this by having stronger lateral interactions. In this specific system the contrast between the P and AP structures is not limited to the *β*-sheet symmetry, the two also differ in the relative arrangement of unit cells with the P structure having a herringbone (or parquet) pattern which buries less surface per lattice plane in the early stages of lateral growth, even though this growth is ultimately more isotropic and stronger.

Quasi-2D aggregates, those with a cross-sectional aspect ratio far from 1, are known to readily form helical ribbons which are then geometrically hindered from hierarchical assembly, leading to slower kinetics, thereby slowing or limiting aggregation^11^. We are now obliged to add a counterexample where the anisotropic type of lateral growth which leads to ribbons and fibrils more than than to microcrystals may overall slow the kinetics relative to 3D growth, but where it is still better for a given polymorph to be growing laterally than to stay longer at the stage of pure 1D aggregation. This case of early anistotropic lateral growth leading to eventual dominance is relevant in the context of competition for monomers against other polymorphs with a longer lag phase.

In this study we have worked to understand kinetics by quantitatively following the route *structure* → *energetics* → *kinetics*, however it is feasible to build intuition such that the lattice parameters can directly suggest the conditions which will favour or disfavour a given aggregation scheme. The picture which now emerges is that strong lateral interactions are necessary in order to have a fast assembly kinetic, and that lateral interactions must be of roughly equal strength (suggested by roughly equal-sized lattice planes) in order to have isotropic aggregates which will ultimately dominate the aggregation process and progress to form a large amount of precipitate.

Amyloid kinetics are multifaceted. Before the formation of amyloid, oligomeric or disordered droplet assemblies may or may not form, depending on the sequence and solution, and these may compete with *β*-structured assemblies, or seed them, or mature into them^29^. Once *β*-sheet has been formed, even within a quasi-1D paradigm distinctions can be drawn between elongation following unconnected nucleation events, nucleation plus secondary nucleation, and self-seeding by fragmentation; and these distinctions have measurable consequences to the kinetic^30^. This quasi-1D approximation should allow meaningful investigation into the aggregation kinetics in particular at low concentrations, where all fibrils must nucleate to a finite thickness in order to be stable, but few fibrils will grow to much greater thickness than that required. A 1D picture is also trivially valid in the case that the chain has steric, electrostatic, or other constraints which prevent assembly in higher dimensions.

With increasing concentration or increasing interaction strength, reduced free energy barriers allow lateral assembly either hierarchically (as modelled in the present work) or *via* secondary nucleation of new *β*-sheets at the surface of existing sheets^31^. The resulting intermediate-dimensionality extended structures, between pseudo-1D fibrils and pseudo-infinite 3D crystals may be present in various competing polymorphic structures and shapes, here we have found and discussed an example in which differing polymorphs compete with each other, preventing or delaying dominance of the more thermodynamically stable polymorph over a wide range of conditions.

## Methods

### Molecular simulation

To relate the crystallography to the nanocrystallites studied *via* solution X-ray, an atomistic model nanocrystal of 1296 peptides (roughly cubic, dimension ≈10 nm or 6 × 6 × 18 two-peptide steric zippers) was built following the **4R0P** crystal structure, and immersed in a periodic box of TIP3P atomistic water^32^. The structure was thermalised and allowed to relax for 15 ns in a simulated aqueous environment using the AMBER molecular dynamics system^33^ and ff14SB forcefield^34^ without showing any major reordering. Scattering was calculated by an orientationally averaged Fourier transform using CRYSOL^35^. Figure (1(b)) shows that the calculated scattering curve is compatible with the solution WAXS spectrum. Four unit cells of the crystal structure are shown in Fig. 1(d). The translational unit cell parameters are *a* = 43.2 Å, *b* = 19.6 Å and *γ* = 90°, however if lattice transforms including rotations and translations are permitted, the *a* cell length becomes 21.6 Å.

### Desolvation energy calculation

To understand the anisotropic growth kinetics of the competing P and AP structures, free energies per buried peptide to desolvate a lattice plane Δ*G*_*a*_°, Δ*G*_*b*_°, Δ*G*_*c*_° were calculated, and also Δ*G*_*zip*_° to bury a steric zipper. Within a linear approximation, the free energy cost to break an interface for example in *a* should be writeable in the form:

3 $$\Delta G={n}_{b}{n}_{c}\Delta {G}_{a}^{\circ }.$$

This linear approximation should become increasingly valid for larger aggregates, as edge effects, cooperativity and finite-size thermodynamics become progressively less significant. The measured values for Δ*G*_*a*,*b*,*c*,*zip*_° therefore depend to some extent on the size of the blocks which are broken or joined in order to evaluate them. In order to have more accurate free energies close to the decisive region of small initial aggregates, capturing cooperativity at approximately the right lengthscales 20–40 Å, block size combinations for each interface were chosen as follows, where *G*_*i*,*j*,*k*_ is the calculated reference free energy for a block of size *i* × *j* × *k*:

4 $$\Delta {G}_{c}^{\circ }=({G}_{1,1,10}-10{G}_{1,1,1})/9$$

5 $$\Delta {G}_{zip-P}^{\circ }=({G}_{1,2,10}-2{G}_{1,1,10})/9.5$$

6 $$\Delta {G}_{zip-AP}^{\circ }=({G}_{1,2,10}-2{G}_{1,1,10})/10$$

7 $$\Delta {G}_{b}^{\circ }=({G}_{2,4,3}-2{G}_{2,2,3})/6$$

8 $$\Delta {G}_{a}^{\circ }=({G}_{2,4,3}-2{G}_{1,4,3})/12.$$

Each *G*_*i*,*j*,*k*_ is found as an average over 50 blocks cut from the large nanocrystal MD simulations of P or AP IFQINS structures already used to calculate scattering. Individual blocks are minimised in a Generalised Born solvent model^26^ and the converged energies averaged. The *P* zipper energies (Δ*G*_*zip*−*P*_°) were found by joining two sheets of ten peptides and then dividing by only 9.5 because of the 0.5*c* overhang in the *c* axis between adjacent sheets defined by the deposited crystal structure. As discussed under the assembly thermodynamics (Eq. 2), the buried area for interfaces is then scaled up again by *n*_*c*_−0.5 for the P structure rather than by *n*_*c*_ as for the AP structure, thus recovering the original measured Δ*G* for the measured interface sizes.

### Kinetic rate equation network

The model for self-assembly of the peptides was defined as a system of *N* rigid bodies each with six faces labelled *a*+, *a*−, *b*, *zip*, *c*+, *c*−. Collision rates were calculated for the peptides (and assemblies of them) based on the equations for diffusion coefficients *D* of rod-like particles due to Ortega and de la Torre^36^. Any coupling between orientation and direction of movement was ignored such that collisions were resolved based on the surface area of the assembly-competent planes, determined from the crystal lattice. In this form an example collision rate constant for two rectangular crystallites of shape *i*, *j*, *k* and *u*, *v*, *w* matching *a*+ and *a*− planes such that *v* = *j* and *w* = *k* is:

9 $${k}_{a+a-}(u,v,w,i,j,k)=2{e}^{-3}(D(u,v,w)+D(i,j,k))\sqrt{2bc\cdot jk}.$$

The barrier term e^−3^ is assigned based on the loss of translational and rotational degrees of freedom on joining two peptide blocks, calorimetric studies have found that the true barrier for small peptides to assemble is indeed of the order 2–5 *k*_*B*_*T* at 300 K^37^. For assembly of larger peptides and proteins an energetic cost to unfold should be added to the barrier term for monomer collisions, for example a barrier of 10.1 *k*_*B*_*T* (25 kJ/mol) is quoted for full-length human lysozyme.

Number density of given species in the simulation volume enters the rate equation directly, with no account made of any spatial correlations in the solution:

10 $${r}_{a+a-}={k}_{a+a-}{N}_{ijk}{N}_{uvw}/V.$$

In the case of homodimerisation, such that *ijk* = *uvw*, the symmetry factor *N*_*ijk*_*N*_*uvw*_ is replaced by *N*_*ijk*_(*N*_*ijk*_ − 1)/2. In the case that two monomers collide, selection of P or AP geometry was made with a 50% chance for each. Once a monomer in an assembly was committed to P or AP, it could return to an uncommitted state only by leaving the assembly. This model does not allow for heterogenous nucleation of P fibrils from AP, or for formation of mixed P/AP fibrils: P and AP structures interact only indirectly, by competing for monomers. Heterogenous nucleation may be added in future iterations of the research.

To define Arrhenius-like rates for a given aggregate to split, it is necessary to set a dynamical timescale. For example the rate for aggregates of a given geometry *u*, *v*, *w* to split on some *c* plane is set as:

11 $${r}_{c}(u,v,w)={k}_{c}(u,v){N}_{uvw}(w-1)$$

12 $${k}_{c}(u,v)=\frac{1}{{\tau }_{0}}\exp [uv\Delta {G}_{c}^{\circ }/{k}_{B}T]$$

where *τ*_0_ is chosen as the time for a single peptide to diffuse its own length.

**Figure 6:**
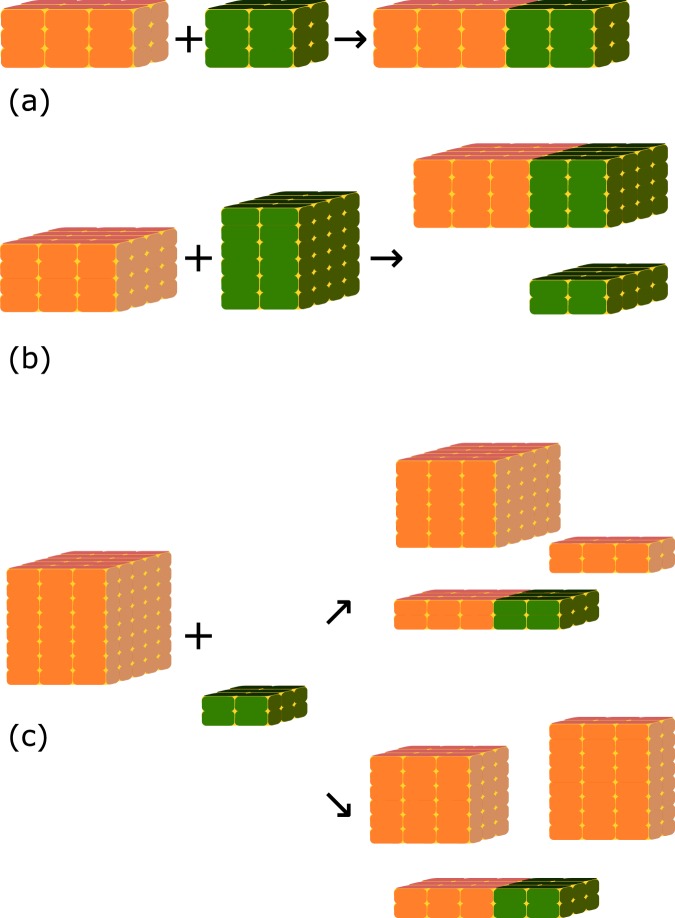
Illustration of **(a)** a simple collision **(b)** a collision with a split and **(c)** a collision with two choices of cleavage planes, resulting in different rates for the overall multistep reaction. [Graphics produced using inkscape 0.91 http://inkscape.org].

The above system allows a rate equation network for collision of rectangular objects which have at least one matching face to be constructed, however such a network quickly and unphysically leads to three populations of aggregates which are extended in each of the lattice axes, and which have zero rates to combine between populations. In order to control complexity of the calculation it was not feasible to track the full space of non-rectangular aggregates, however these were treated ‘virtually’ by allowing complex collisions including a splitting process into the rate, such that objects with only one or zero matching dimensions could still collide, and the final state after the reaction would contain again only rectangular agggregates. Figure 6 illustrates the multistep reactions treated. Figure 6(a) corresponds to the single-step collision of Eq. 10, while for Fig. 6(b) we combine rate constants for joining (*k*_*a*+*a*−_) and splitting (*k*_*c*_):

13 $${r}_{a+a-}={\tau }_{0}{k}_{c}(2,4){k}_{a+a-}(3,4,4,2,4,6)\frac{{N}_{3,4,4}{N}_{2,4,6}}{V}.$$

The two-to-three body process of Fig. 6(c) allows a choice of two pairs of cleavage planes given a collision surface of two non-matching bodies. To manage the complexity of the calculation, rates for each choice of planes were calculated, and only the fastest one retained in the kinetic system. Any process with *k* < 10^−50^ was also automatically discarded from the rate equation system.

The rate equations for single collisions and collision-plus-split are balanced by simple splitting for single collisions, and by the reverse two body process for the collision-plus-split, however no three-to-two process was constructed as a simple calculation of likely rates for this found extremely small values. The two-to-three process also had extremely low rates, but was retained in order to avoid pathological situations such that a 10 × 12 × 102 aggregate could not ever assemble with a 9 × 10 × 101 aggregate.

Given the set of rate equations described above (a ‘kinetic master equation’) it should be possible to make an analytical statement of the non-equilibrium kinetics and the final equilibrium state of the system^38^. Such analyses typically do not capture stochastic effects such as nucleation, which are often important for amyloid formation, so instead the decision was made to sample the rate equation set numerically using the event-driven Gillespie algorithm^39^. In this method, the rates for all possible forward or backward processes given the current state of the system are calculated, and a single process to carry out is then selected randomly with a weight proportional to the rate for that process. The system is then updated according to the reaction chosen, and the rates re-calculated with re-use of information from the previous iteration. Rate constants for given reactions are calculated only once, on the first occasion that given reactants are added to the system, and then cached so that future rate calculations for that reaction can be made cheaply. For each simulation system, 6 million peptides were used, and concentration was controlled by setting the volume *V*.

### Experimental methods

Material from the same batch of IFQINS as previous works^9,11^ was used, and other experimental details were set to be consistent with these previous studies wherever possible.

The IFQINS was made by solid phase peptide synthesis using the Wang resin support and O-(benzotriazole-1-yl)-1, 1, 3, 3-tetramethylcarbamide tetrafluoroborate (TBTU) as the coupling reagent. The base was N, N’ diisopropylethylenamine. Intramolecular cyclisation was avoided by the addition of 1-Hydroxybenzotriazole (HOBt). After swelling the resin overnight in Dimethylformamide (DMF), TBTU (4 equiv), Fmoc-protected amino acid (4 equiv), DiPEA (4 equiv) and HOBt (4 equiv) also in DMF were added and shaken. A time of one hour was allowed for coupling, after which the resin was washed with DMF (4 × 1 min) and DCM (4 × 1 min). The Fmoc group was removed using piperidine (15 min). The peptide was cleaved for 1 h at 0 °C from the resin using HF and 10% anisole. The peptide was then precipitated with anhydrous tert-butylmethyl ether, dissolved in AcOH, and lyophilized. The lyophilate was further purified *via* RP-HPLC with gradients of water and acetonitrile. The molecular weight was measured as 720.8 Da, consistent with the expected mass of 720.82 Da.

At the beamline, lyophilized IFQINS was mixed with MilliQ water at either 1.5 mM or 5 mM then left for 24 hours to allow initiation of self-assembly. After 24 h, WAXS was carried out on the evolving peptide solution.

Scattering was performed at room temperature at the SAXS/WAXS beamline of the Australian synchrotron. The experiments used a beam of wavelength of *λ* = 1.03320 Å (12.0 KeV) with dimensions 300 *μ*m × 200 *μ*m and a typical flux of 1.2 × 10^13^ photons per second. Data was collected at at *q* ranges between 0.03–1.5 Å^−1^, although only *q* ranges 0.3–0.7 Å^−1^ were found to contain useful signal, the high-*q* being noisy and the low-*q* being dominated by form factor, which is uncontrolled in the experiment due to the wide variety of aggregate sizes and morphologies present. Samples were loaded into a 96 well plate on a robotically controlled x-y stage, then transferred to the beamline via a quartz capillary connected to a syringe pump. A Pilatus 1M detector was used to record 2D diffraction, which was then rotationally averaged to create a 1D signal. Spectra were recorded under flow (0.15 ml min^−1^) in order to prevent beam damage to the sample. Fifteen replicate spectra were recorded, the averaged spectra are shown after background subtraction against MilliQ water in the same capillary.

Tapping-mode force microscopy images were collected in air using a Multimode VIII (Bruker, USA) Atomic Force Microscope (AFM), and a Nanoscope V controller (Bruker, USA). Areas of approximately 3 μm^2^ were scanned using Antimony (n) doped silicon cantilevers with a spring constant of 40 N/m (RTESPA-300, Bruker) and resonant frequency of approximately 300 kHz. The resolution of recorded images was 512 × 512. All scans were flattened (first order) in the manufacturer’s supplied Nanoscope 8.15 analysis software and no further image processing was applied. 

Calculations made use of the University of Luxembourg HPC facility^40^ and molecular graphics were prepared using pymol^41^.

## Data Availability

The software and structures discussed are available from the corresponding author on request.
